# Sprouty2 regulates positioning of retinal progenitors through suppressing the Ras/Raf/MAPK pathway

**DOI:** 10.1038/s41598-020-70670-2

**Published:** 2020-08-13

**Authors:** Jian Sun, Jaeho Yoon, Moonsup Lee, Yoo-Seok Hwang, Ira O. Daar

**Affiliations:** Cancer and Developmental Biology Laboratory, National Cancer Institute, Frederick, MD 21702 USA

**Keywords:** Cell biology, Developmental biology

## Abstract

Sproutys are negative regulators of the Ras/Raf/MAPK signaling pathway and involved in regulation of organogenesis, differentiation, cell migration and proliferation. Although the function of Sproutys have been extensively studied during embryonic development, their role and mode of action during eye formation in vertebrate embryonic development is still unknown. Here we show that *Xenopus* sprouty2 is expressed in the optic vesicle at late neurula stage and knockdown of Sprouty2 prevents retinal progenitors from populating the retina, which in turn gives rise to small eyes. In the absence of Sprouty2, progenitor cell population of the retina can be restored by blocking the MAPK signaling pathway through overexpression of DN-Ras or DN-Raf. In contrast, activation of the MAPK pathway through overexpression of a constitutively active form of c-Raf (ca-Raf) inhibits progenitor population of the retina, similar to the Sprouty2 loss-of-function phenotype. Moreover, we present evidence that the retinal defect observed in Sprouty2 morphants is attributed to the failure of proper movement of retinal progenitors into the optic vesicle, rather than an effect on progenitor cell survival. These results suggest that Sprouty2 is required for the positioning of retinal progenitors within the optic vesicle through suppressing Ras/Raf/MAPK signaling pathway.

## Introduction

A series of gradually restrictive phases are required to properly form the vertebrate retina and to control the available cell fates. The formation of the retina can be considered to occur in three progressive stages starting with forming the eye field, followed by the optic vesicle and finally the optic cup stages. Just subsequent to gastrulation, the eye field forms with the specification via transcription factors of the anterior neural plate region which will ultimately form the retina^1^. During the neurula stage, the neural plate bends upwards to form the neural tube, the eye field tissue evaginates to create optic vesicles on both sides of the neural tube. These optic vesicles will contact the overlying ectoderm, initiating invaginations resulting in the formation of the optic cup where the lens will form^2^. The eye field and optic vesicles are comprised of proliferating retinal progenitor cells.

A critical step in the formation of the developing retina is to position retinal progenitors within the eye field, where local signals within the niche will direct their eventual fate^3,4^. Three major morphogenetic movements are required to drive retinal progenitors to be correctly positioned in the retina. The dorsal ectoderm is located within the anterior signaling centers during gastrulation, and through epiboly movements the anterior ectoderm will arise. At the neural stage, cells from the anterior neural ectoderm disperse and intermix to form the basic retinal progenitor pool within the eye field. Subsequently, the retinal progenitor cells move bilaterally and increase to form eye primordia^5,6^. Membrane localized as well as secreted signaling molecules play important roles in forming the eye field. For example, Wnt11 promotes eye field development by regulating cell cohesion and dispersal within the eye field through antagonism by locally secreted canonical Wnt^7^. Ligand-induced activation of the fibroblast growth factor receptor (FGFR) regulates ephrinB1 signaling that modulates the positioning of retinal progenitor cells within the eye field^8^. EphrinB1 signaling through the PCP pathway during eye field formation^9^ is mediated by an interaction with Dishevelled. Upon FGFR activation, ephrinB1 is tyrosine phosphorylated, disrupting the association between Dishevelled and ephrinB1^10^.

Downstream of receptor tyrosine kinases, such as the FGFR, are Sprouty proteins, which are negative intracellular regulators of Ras/Raf/MAPK (ERK) signaling^11,12^. In Drosophila, Sprouty functions as an antagonist of FGF signaling during tracheal branching^13^. Four vertebrate Sproutys, Sprouty1–4, have been identified and have been shown to function throughout embryonic development^13^. In *Xenopus*, regulation of the duration of ERK activity by Sprouty2 contributes to dorsoventral patterning^14^. In mice, Sprouty1 mutation induces hyperactivation of the ERK in the Wolffian duct with increased ectopic branching morphogenesis^15^. Sprouty2 is essential for establishing the cytoarchitecture of the auditory sensory epithelium by antagonism of FGF signaling^16^. Sprouty2 and Sprouty4 serve to block FGF-mediated ERK activation to regulate diastema tooth formation in mouse^17^. In the brain, Sprouty1 and Sprouty2 repress FGF-ERK signaling from the rostral patterning center to regulate cortical development^18^. These results establish Sproutys as critical modulators of ERK signaling during organogenesis. Interestingly, mice null for both Sprouty4 and Sprouty2 show substantially more severe defects in the eye when compared to single Sprouty4 null mice, which exhibit mild eyelid closure defects^19^. Thus, Sprouty2 is likely to have a significant role in eye development. Although it has been shown in Drosophila that mutations in Sprouty2 display excess photoreceptors, cone cells, and pigment cells^20^, the role of Sprouty2 in vertebrate eye development is still largely unknown.

Here we take advantage of targeted injections into the major retina precursor blastomeres and use the *Xenopus* eye as a tractable model for understanding how Sprouty2 regulates eye formation in *Xenopus*. We present evidence that excess activity of MAPK blocks retinal progenitors from populating retina and that Sprouty2 is required for retinal progenitors to properly position within the optic vesicle by suppressing Ras/Raf/MAPK signaling.

## Results

### *Xenopus* Sprouty2 is required for normal eye development

**Figure 1:**
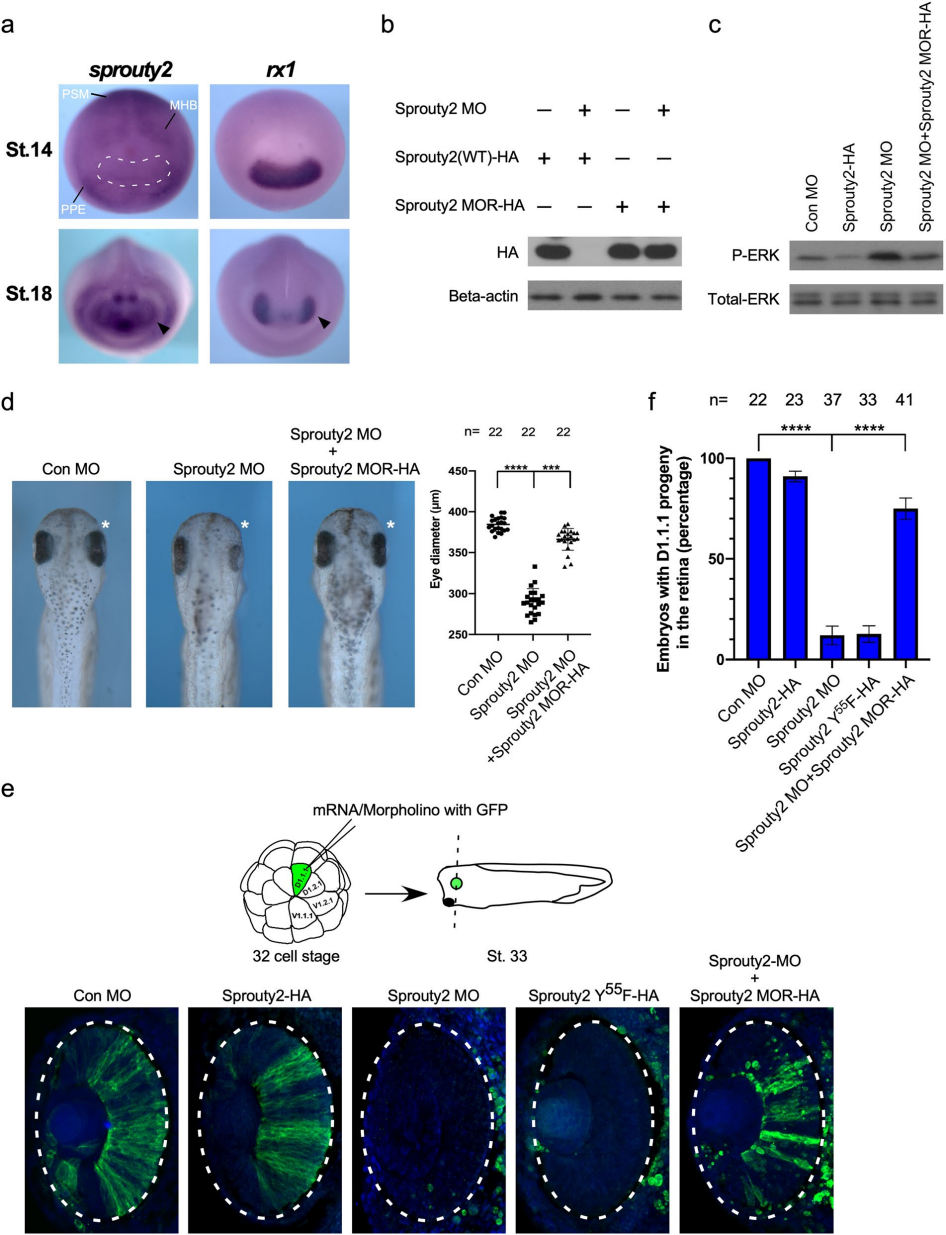
Sprouty2 is required for eye development through regulating the population of retina. (**a**) Whole-mount in situ hybridization reveals that *sprouty2* is expressed in the optic vesicle at late neurula stage. Black arrowhead indicates optic vesicle on stage 18 embryo. The eye field was outlined with white dotted line. (**b**) Embryos were injected with wild type Sprouty2 mRNA (WT) or morpholino-resistant (MOR) form of Sprouty2 alone, or plus Sprouty2 morpholino at one cell stage, and analyzed by Western blot at late gastrula stage. Sprouty2 MO blocks the expression of Sprouty2 WT but not the Sprouty2 MOR. (**c**) Embryos were injected with Sprouty2 WT RNA or sprouty2 MO or a combination of Sprouty2 MO and Sprouty2 MOR RNA at one cell stage. Phosphorylation of ERK was analyzed by Western blot using embryo lysate from late gastrula stage. (**d**) D1.1.1 blastomere was injected with Sprouty2 MO with or without Sprouty2 MOR RNA, and the eye size was analyzed at stage 36. Dorsal view of the eyes in Sprouty2 morphant shows a significant reduction in diameter which could be well rescued by Sprouty2 MOR RNA co-injection. Quantification of eye diameter with one-way ANOVA (Dunnett’s multiple comparison), ****P < 0.0001, ***P < 0.001, Error bars indicate ± SD. (**e**) The D1.1.1 blastomere was injected with GFP mRNA plus Sprouty2 WT or Sprouty2Y^55^F RNA alone or Sprouty2 MO with or without Sprouty2 MOR RNA. Embryos were sectioned and immunostained with GFP antibody (Green) on the eye region at stage 33. The retina was outlined with an oval dotted line. (**f**) The Histograms indicate the percentage of embryos with D1.1.1 progeny in the retina from three biological repeats. Quantification with one-way ANOVA (Sidak's multiple comparison), ****P < 0.0001, Error bars indicate ± SD.

Since loss of Sprouty2 in a Sprouty4 null mouse background caused severe eye development defects^19^, we focused on the function of Xsprouty2 in eye development. Whole-mount in situ hybridization indicated that at the early neurula stage, the expression of *Xsprouty2* was detected at the mid-hindbrain boundary (MHB), in pre-placodal ectoderm (PPE) and presomitic mesoderm (PSM) but not the eye field (Fig. 1a). The expression of eye field marker *rx1* was used for comparison. At the late neurula stage, expression of Xsprouty2 was clearly expressed in the optic vesicle (Fig. 1a). To examine the loss of Sprouty2 phenotypes during eye development, a translation blocking morpholino (MO) for Sprouty2 was designed. The blocking efficiency of this morpholino was tested by Western blot of lysates from embryos exogenously expressing wild type Sprouty2 or a morpholino-resistant (MOR) form of Sprouty2 harboring substitutions in 5 wobble codons (Fig. 1b). Sprouty2 has been reported to function as a negative regulator for MAPK in both tissue culture and embryos^18,21,22^. To further confirm the morpholino efficiency in vivo, we examined MAPK activity by Western analysis using phospho-specific MAPK antibody. Overexpression of Sprouty2 caused a significant decrease in activated phosphorylated MAPK, while injection of Sprouty2 MO substantively increased the amount of phosphorylated MAPK. This MO-mediated increase could be suppressed by co-injection of Sprouty2 morpholino-resistant RNA (Fig. 1c), which suggests that Sprouty2 MO efficiently blocks endogenous Sprouty2. To examine whether loss of Sprouty2 affects eye development, we performed target injection of Sprouty2 MO into the D1.1.1 blastomere, a major contributor (> 50%) to the retina^8^ at the 32-cell stage. In embryos, the Sprouty2 MO injected side displayed a much smaller eye when compared to the uninjected side, and this phenotype is rescued by co-injection of Sprouty2 MO resistant RNA (Fig. 1d). These data suggest that Xsprouty2 is required for normal eye development.

### Sprouty2 regulates retina population through suppressing Ras/Raf/MAPK signal

It has been shown that fibroblast growth factor (FGF) modulates ephrinB1/Dishevelled (Dsh2) interactions to regulate the positioning of retinal progenitor cells within the definitive eye field^8–10^. Sprouty2 has been reported to inhibit FGF-mediated gastrulation movements through repressing Ca^2+^ and PKCδ signaling in the frog embryo^23^. Therefore, we tested whether Sprouty2 also plays a role in the positioning of retinal progenitors within the retina. Sprouty2 RNA or morpholino was injected with GFP RNA into the D1.1.1 blastomere and embryos were cultured until stage 33. Control embryos showed normal retina population (GFP positive signal), and over expression of Sprouty2 did not affect this outcome. In contrast, MO-mediated knockdown of Sprouty2 or overexpression of a dominant negative form of Sprouty2 (Sprouty2Y^55^F) significantly inhibited population of the retina by progenitor cells (Fig. 1e,f). Co-injection of Sprouty2 morpholino-resistant RNA rescued the phenotype in the Sprouty2 morphants (Fig. 1e,f), indicating a requirement for Sprouty2 in regulating D1.1.1 progeny positioning within the retina.

**Figure 2:**
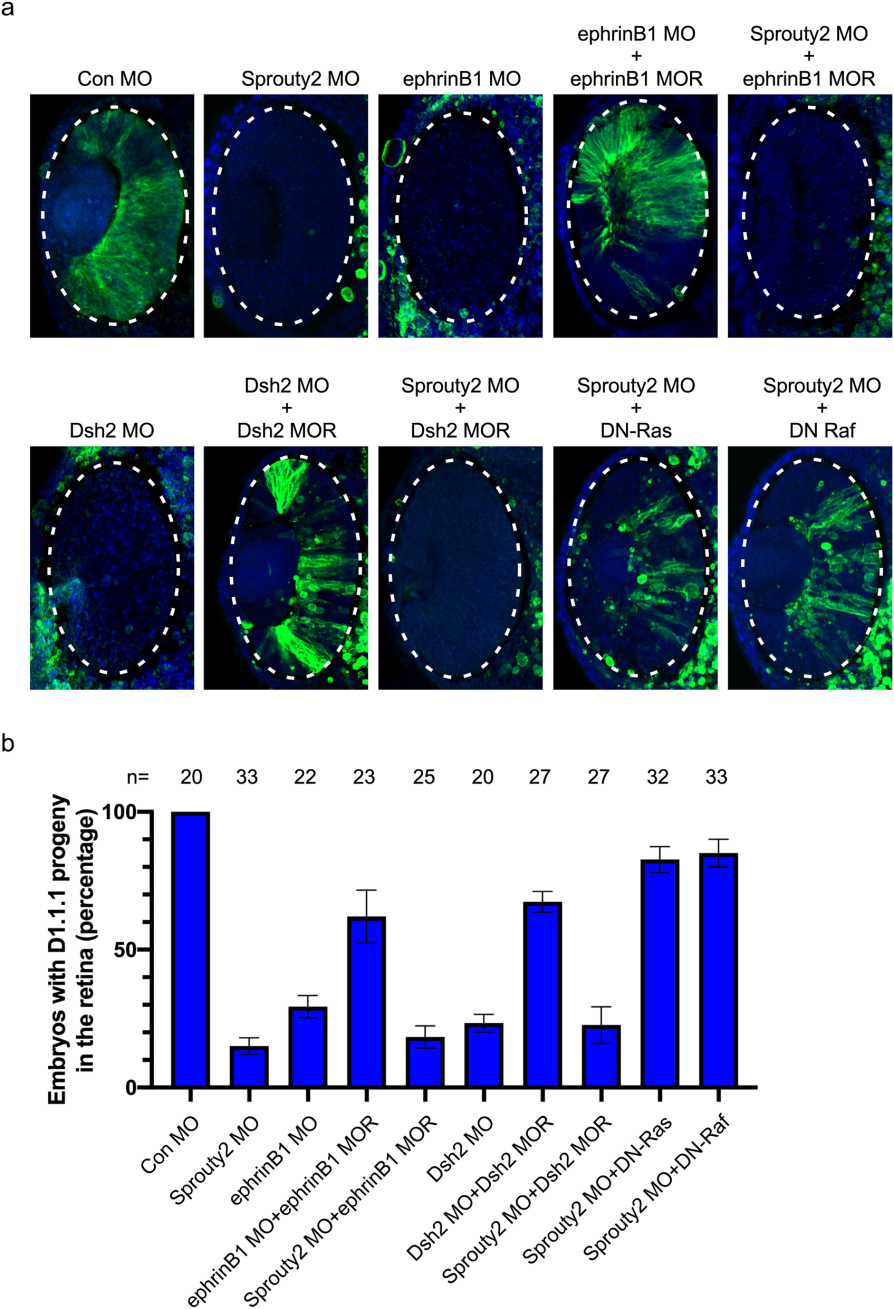
Blocking of Ras/Raf/MAPK pathway rescues Sprouty2 MO-induced block to retina population. (**a**) Indicated morpholinos or RNAs were injected with GFP RNA into D1.1.1 blastomere at 32 cell stage. Embryos were then sectioned and immunostained with GFP antibody (Green) at stage 33. Images were taken of the eye region. The retina was outlined with an oval dotted line. (**b**) Histograms represent the percentage of embryos with D1.1.1 progeny (GFP positive signal) within the retina from three biological repeats. Quantification with one-way ANOVA (Sidak's multiple comparison), *P* < 0.0001, Error bars indicate ± SD.

Previous studies have demonstrated that ephrinB1 signals via its intracellular domain to control retinal progenitor movement into the *Xenopus *eye field by interacting with Dsh2 and co-opting the planar cell polarity (PCP) pathway^9^. To examine whether Sprouty2 regulates positioning of retinal progenitors through the ephrinB1/Dsh2 mediated PCP pathway, we co-injected either ephrinB1 or Dsh2 RNA along with Sprouty2 MO into the D1.1.1 blastomere. Although ephrinB1 MO-resistant RNA and Dsh2 MO-resistant RNA were able to rescue their respective morphants, neither MO-resistant RNA could rescue the Sprouty2 MO-mediated block to retinal population (Fig. 2a,b). Since Sprouty2 is known to inhibit the Ras/Raf/MAPK pathway in tissue culture cells and embryos, we tested the possibility that Sprouty2 may regulate retinal progenitor positioning via suppressing MAPK activity. Indeed, injection of DN-Ras or DN-Raf RNA into the D1.1.1 blastomere significantly restored the retinal population in the absence of Sprouty2 (Fig. 2a,b). These results suggest that Sprouty2 likely regulates retinal progenitor positioning through suppressing the Ras/Raf/MAPK pathway, but it is independent of the ephrinB1/Dsh2 mediated PCP pathway.

### Ras/Raf/MAPK signal negatively regulates retina population

**Figure 3:**
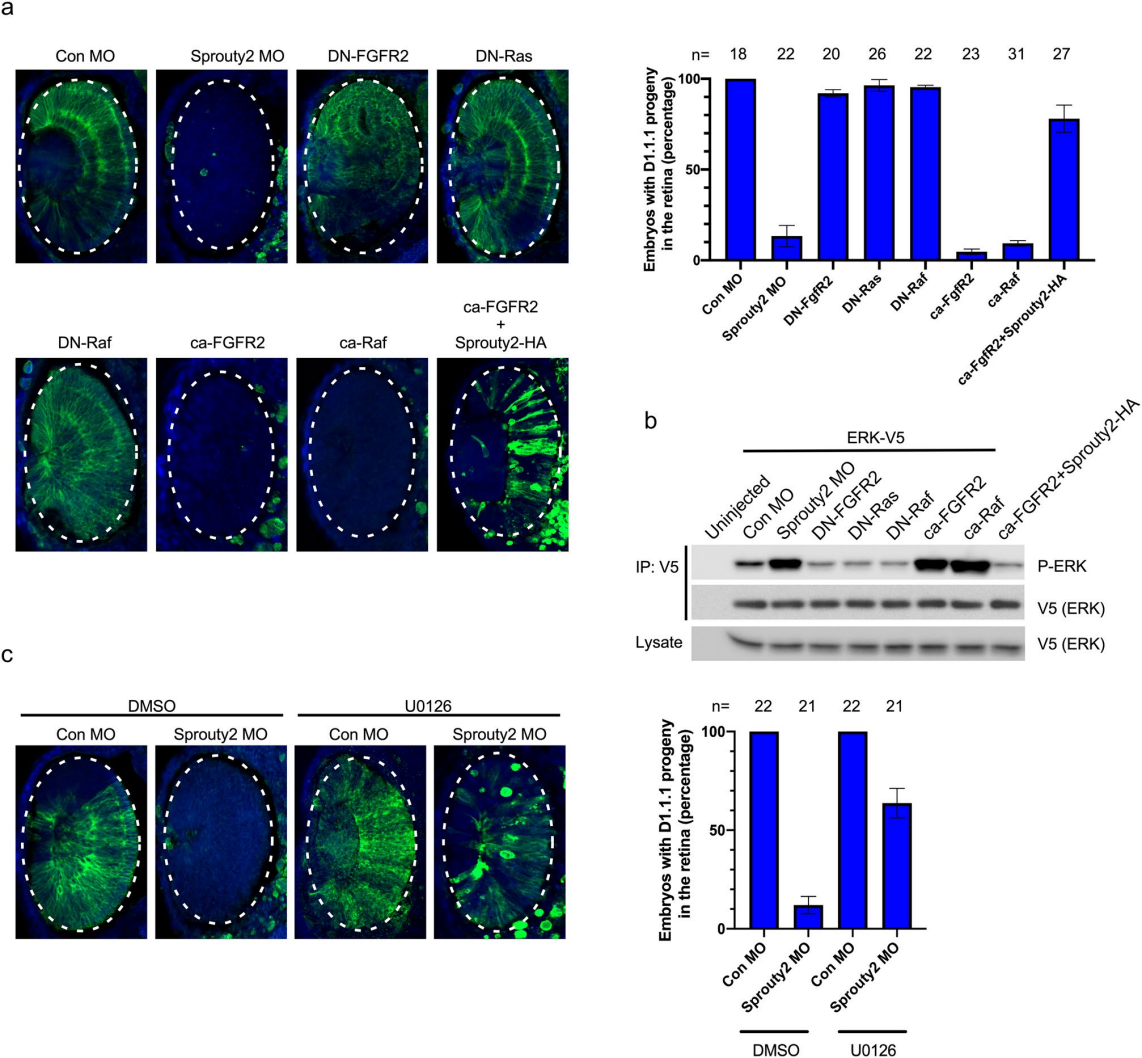
Over-activity of MAPK inhibits positioning of retinal progenitors within retina. (**a**) The indicated morpholinos or RNAs were injected with GFP mRNA into D1.1.1 blastomere at 32 cell stage. Embryos were then sectioned and immunostained with GFP antibody (Green) at stage 33. Images were taken of the eye region. The retina was outlined with an oval dotted line. Histograms represent the percentage of embryos with D1.1.1 progeny (GFP positive signals) within the retina from three biological repeats. Quantification with one-way ANOVA (Sidak's multiple comparison), *P* < 0.0001, Error bars indicate ± SD. (**b**) Embryos were injected with RNAs or Morpholinos along with ERK-V5 RNA into D1.1.1 blastomere at 32 cell stage and lysed at late neurula stage for immunoprecipitation with v5 agarose beads. Precipitates were then immunoblotted with phospho-ERK and total ERK antibodies. Three independent repeats were performed. (**c**) D1.1.1 blastomere was injected with control MO or Sprouty2 MO along with GFP mRNA at 32 cell stage. The injected embryos were either treated with DMSO or U0126 from late gastrula to late neurula stage. Embryos were then sectioned and immunostained with GFP antibody (Green) at stage 33. Histograms represent the percentage of embryos with D1.1.1 progeny (GFP positive signals) within the retina from three biological repeats. Quantification with one-way ANOVA (Sidak's multiple comparison), *P* < 0.0001, Error bars indicate ± SD.

To further investigate how MAPK activity could affect retinal progenitors positioning within the retina, we modulated MAPK activity in the D1.1.1 blastomere. The MAPK pathway was activated by injection of an activated form of the FGFR (ca-FGFR2) or an activated Raf (ca-Raf), which markedly inhibited the D1.1.1 progeny from populating the retina, similar to the Sprouty2 knockdown phenotype (Fig. 3a,b). The introduction of DN-Ras or DN-Raf blocks the MAPK pathway, but failed to affect retinal population by D1.1.1 progeny (Fig. 3a,b). Interestingly, co-injection of Sprouty2 RNA successfully rescued the ability of the D1.1.1 progeny to enter the retinal field in the presence of the ca-FGFR2, suggesting Sprouty2 may function downstream of FGFR2 (Fig. 3a,b). Furthermore, treatment with the U0126 MEK inhibitor in the absence of Sprouty2 (due to Sprouty2 MO) partially but substantively rescued D1.1.1 progenitor population of the retina (Fig. 3c). These data suggest that low level of MAPK activity is normally required for retinal progenitors to appropriately position within the retina and enhanced MAPK signaling prevents these progenitors from populating the retina.

### Sprouty2 is required for retinal progenitor movement into the optic vesicle through suppressing MAPK activity

**Figure 4:**
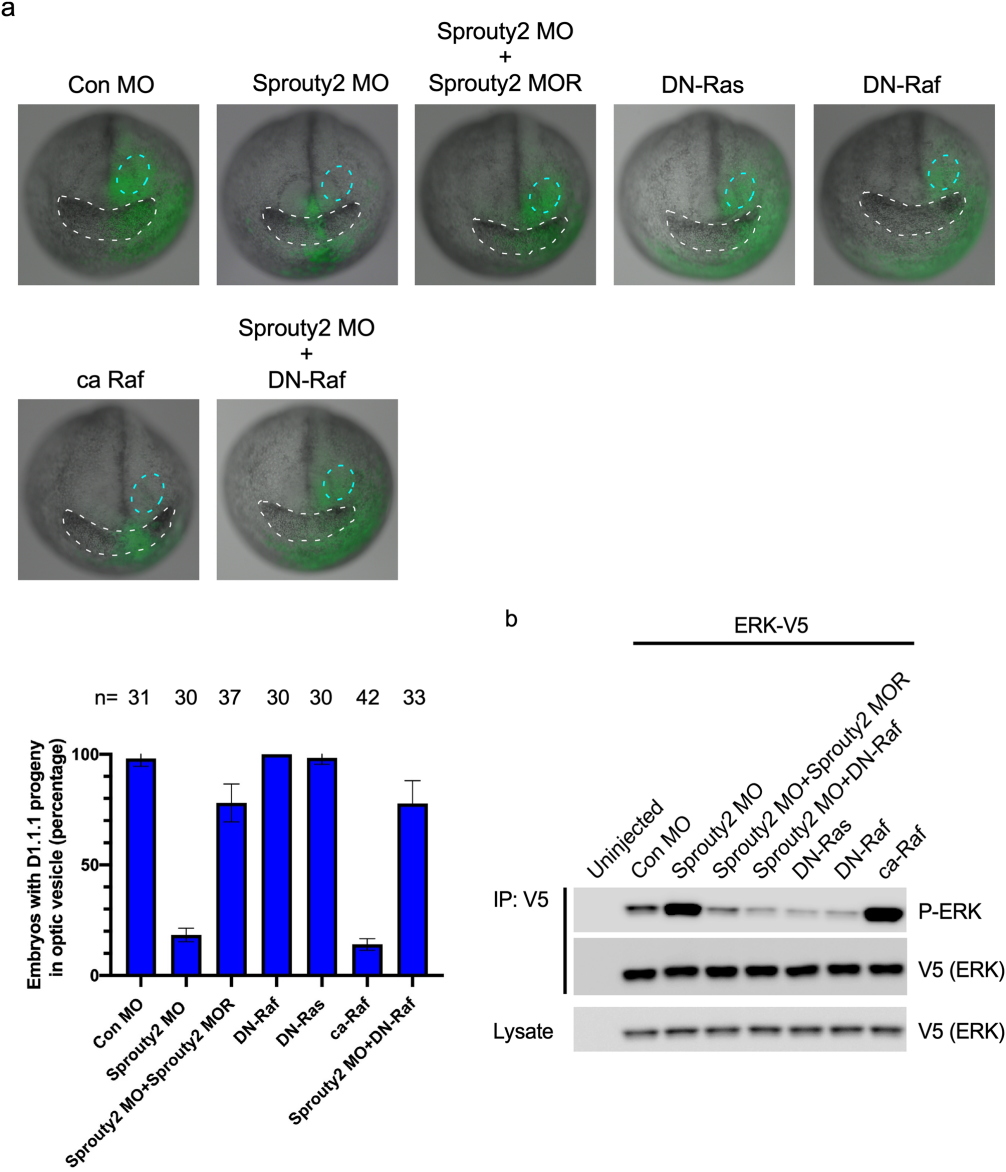
Sprouty2 regulates retinal progenitor movement into optic vesicle through repressing MAPK activity. (**a**) D1.1.1 blastomere was injected with GFP RNA plus RNAs and morpholinos as indicated. Embryos were then harvested and analyzed at late neurula stage. The cement gland was outlined with white dotted line and the optic vesicle was outlined with cyan dotted line. Histograms represent the percentage of embryos with D1.1.1 progeny (GFP positive signals) within the optic vesicle from three biological repeats. Quantification with one-way ANOVA (Sidak's multiple comparison), *P* < 0.0001, Error bars indicate ± SD. (**b**) Embryos were injected with indicated RNAs or morpholinos along with ERK-V5 mRNA into D1.1.1 blastomere at 32 cell stage and lysed at neurula stage for immunoprecipitation with V5 agarose beads. Precipitates were then immunoblotted with phospho-ERK and total ERK antibodies. Three independent repeats were performed.

Since Sprouty2 was expressed in the earliest specified retinal progenitor pool at the late neurula stage (Fig. 1a), we tested whether loss of Sprouty2 affects D1.1.1 progeny movement. GFP RNA was injected as a tracer along with Sprouty2 MO into the D1.1.1 blastomere at the 32-cell stage. Embryos were then harvested and analyzed at both the early and late neurula stage. At the early neurula stage, D1.1.1 clones harboring Sprouty2 MO were widely dispersed across the anterior neural plate including the eye field, similar to Control D1.1.1 clones (Supplementary Fig. 1). However, at the late neurula stage, all control D1.1.1 clones were still broadly dispersed across the anterior neural plate, including the optic vesicle and cement gland region, whereas those harboring the Sprouty2 MO were tightly confined near the midline (Fig. 4a). This phenotype could be noticeably rescued by co-injection of Sprouty2 MO-resistant RNA. To determine whether the Ras/Raf/MAPK signaling pathway is involved in Sprouty2-mediated movement of retinal progenitors, DN-Ras, DN-Raf or ca-Raf RNA was injected into the D1.1.1 blastomere. Inhibition of MAPK activity by injection of either DN-Ras or DN-Raf did not affect D1.1.1 progeny movement into the optic vesicle, although the dispersion of D1.1.1 clones in the cement gland region was affected (Fig. 4a,b). In contrast, activating MAPK by injection of ca-Raf confined D1.1.1 clones to the anterior midline, even in the cement gland (Fig. 4a,b). Interestingly, overexpression of DN-Raf fully restored the dispersal of D1.1.1 progeny cells across the optic vesicle in the absence of Sprouty2 (Fig. 4a). These results suggest that inhibition of MAPK signaling by Sprouty2 is required for D1.1.1 progeny movement into the optic vesicle at the late neurula stage but not at the early neurula stage, which is consistent with the expression pattern of *Sprouty2* (Fig. 1a). Although there was no noticeable loss of GFP positive cells in the presence of Sprouty2 MO or ca-Raf expression, it was formally possible that either apoptosis or inhibition of proliferation may explain the lack of cell dispersal beyond the midline rather than cell movement. To address this possibility, we performed both an assay for cleaved-caspase-3 (apoptosis) and phospho-histone-3 (proliferation) in the late neurula stage embryos. No significant apoptosis was detected in the retinal progenitor (Supplementary Fig. 2), and a slight increase in cell proliferation was found in both the Sprouty2 MO and activated Raf-injected progenitors (Supplementary Fig. 3). However, when embryos were examined at tadpole stages, the mislocalized D1.1.1 progeny harboring a Sprouty2 MO displayed robust apoptosis outside the retina, which could be rescued by either co-injection of Sprouty2 morpholino-resistant RNA or DN-Raf RNA (Supplementary Fig. 4). Thus, the data indicate that a reduction of cell movement occurs at the late neurula stage when MAPK is hyper activated, rather than increased apoptosis or decreased cell proliferation. It is known that potential retinal progenitors need to be positioned within the eye field to receive the local environmental signals that will direct their ultimate fates^24,25^. Those mislocalized D1.1.1 descendants likely failed to adopt a retinal fate, which in turn, resulted in eventual apoptotic cell death when the retina has formed at the tadpole stage.

**Figure 5:**
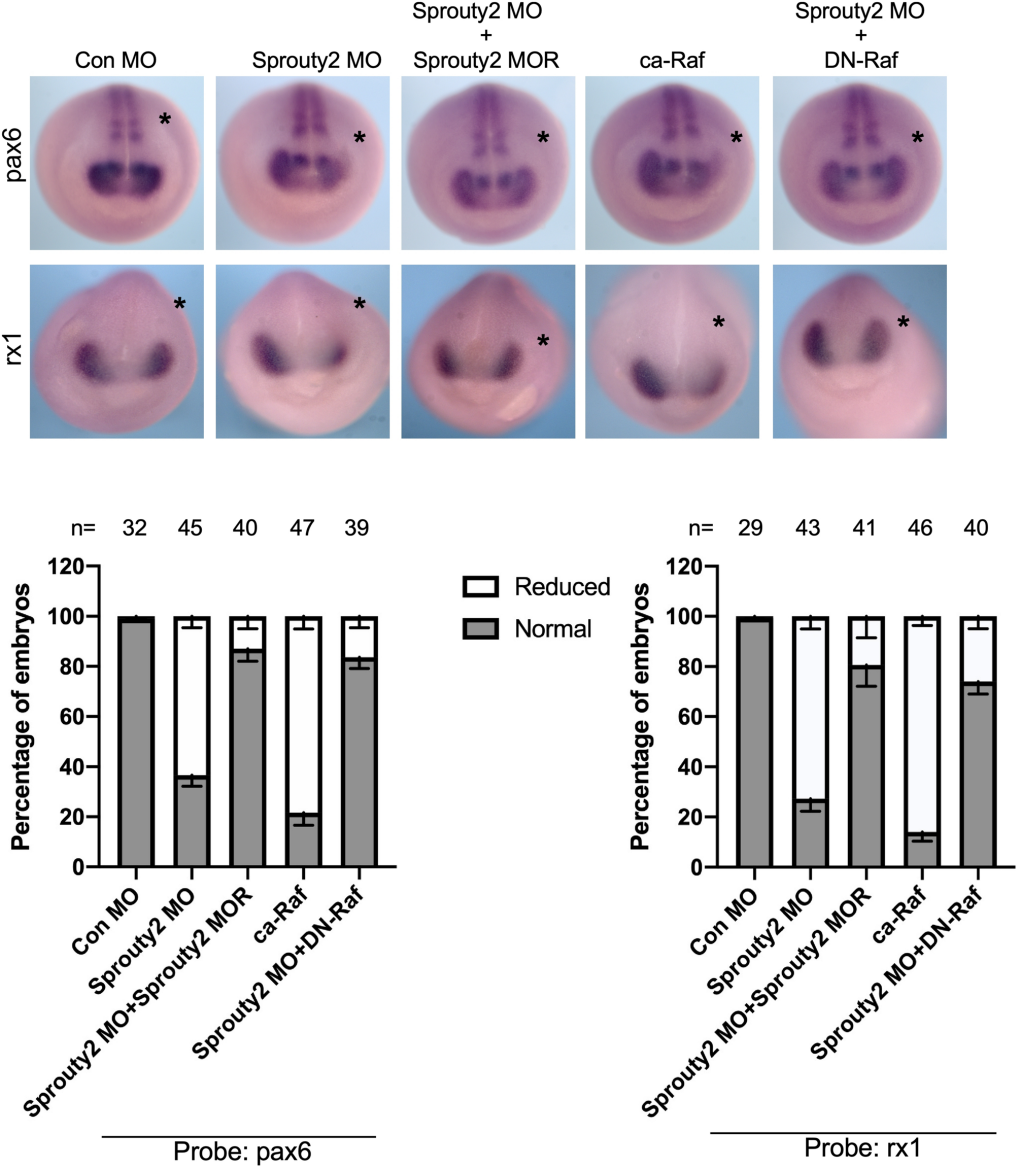
Activation of MAPK signal affects eye specific marker gene expression. D1.1.1 blastomere was injected with RNA or morpholino or a combination of both as indicated. Embryos were then collected at stage 18 and subjected to whole-mount in situ hybridization for eye marker genes *pax6* and *rx1*. Asterisk indicates the injected side. Histograms represent the percentage of embryos with normal or reduced expression of *rx1* or *pax6* from three biological repeats. Quantification with unpaired t test, *P* < 0.001, Error bars indicate ± SD.

Consistent with the failure of D1.1.1 progeny to move into the optic vesicle, the expression of two early eye specific genes, rx1 and pax6, was reduced when Sprouty2 MO was introduced into the D1.1.1 blastomere (Fig. 5). Co-injection of Sprouty2 morpholino-resistant RNA could rescue the expression of *rx1* and *pax6* reduced in Sprouty2 morphants. Introduction of DN-Raf also rescued Sprouty2 MO-induced repression of *rx1* and *pax6* expression (Fig. 5). Activating MAPK by expressing ca-Raf reduced the expression of these two genes, similar to the loss-of-Sprouty2 (Fig. 5). Taken together, these data demonstrate that failure of D1.1.1 progeny to move into the optic vesicle prevents expression of a retinal fate in the absence of Sprouty2.

## Discussion

The retinal precursors are brought to the correct position to form an eye field by a series of morphogenetic movements during gastrulation and neurulation mediated by different signaling pathways such as FGF and Wnt signals^7,8,26^. FGF signaling has been showed to modulate ephrinB1 signaling to regulate the position of retinal progenitor cells within the definitive eye field. Activating FGF signaling by ca-FGFR2 prevents retinal progenitors from populating the presumptive eye field and ephrinB1 over expression can rescue the inhibition of retinal fate caused by FGFR signaling^8^. Further studies showed that ephrinB1 signaling controls retinal progenitor movement into the eye field by interacting with Dsh2 and co-opting the PCP pathway^9^. Thus, FGFR restricts eprhinB1 driven cell movement via the Dsh2/PCP pathway.

FGF also activates multiple intracellular cascades during embryonic development^27,28^. The MAPK/ERK pathway (also known as the Ras–Raf–MEK–ERK pathway) is one of the well-known downstream cascades of the FGFR. However, whether this pathway is involved in regulating the positioning of retinal progenitor within retina is still largely unknown. Sprouty2, a well-studied negative feedback regulator that controls the Ras–Raf–MAPK pathway at multiple levels is an ideal candidate for understanding the role of MAPK pathway in the positioning of retinal precursor cells. Our data shows that *Xenopus* Sprouty2 is expressed in the optic vesicle at late neurula stages. Knockdown of Sprouty2 or overexpression of a dominant negative form of Sprouty2 in the D1.1.1 blastomere inhibits access of D1.1.1 descendants to the retina. Co-injection of either DN-Ras or DN-Raf with significantly restores population of the retina by D1.1.1 progeny cells in the absence of Sprouty2. Excessive activation of the MAPK signal by injection of ca-FGFR2 or ca-Raf prevents D1.1.1 progeny from populating the retina. Co-injection of Sprouty2 rescues the block to retinal population induced by ca-FGFR2 overexpression. By tracking the D1.1.1 descendants’ movement, we found that progeny receiving Sprouty2 MO or expressing ca-Raf are confined to the anterior midline across the cement gland at the late neurula stage. However, all control D1.1.1 clones are broadly dispersed across the anterior neural plate, including the optic vesicle region. Overexpression of DN-Raf fully restored D1.1.1 progeny cell movement into the optic vesicle in the absence of Sprouty2. Thus, we revealed Ras/Raf/MAPK signaling pathway as a negative regulator of retinal precursor positioning within the optic vesicle.

An important step in retinal development is the positioning of progenitors within the eye field where they receive the local environmental signals that will direct their ultimate fate. Only after that step is accomplished, the eye organogenesis occurs^24,25^. Either knock down of Sprouty2 or induction of excessive MAPK activity caused a failure of D1.1.1 descendants to position within the optic vesicle (Fig. 4). These progeny did not adopt a retinal fate and displayed reduced expression of *pax6* and *rx1*. Although these mislocalized D1.1.1 clones did not show increased apoptotic signals at the late neurula stage, they do eventually undergo apoptosis when the retina has formed (Supplementary Figs. 2 and 4). Thus, Sprouty2 is required to sustain retinal progenitors within the optic vesicle, which is necessary for retinal progenitors to receive local environmental signals and express a retinal fate.

Although Sprouty2 has been shown to inhibit gastrulation movements by interfering with FGF-induced PKC signaling as well as protocadherin-mediated PCP signaling^23,29,30^, overexpression of Sprouty2 has no effect on positioning of retinal progenitor cells within the retina (Fig. 1e). Our data indicates that Sprouty2 regulates population of the retina by a PCP pathway independent route. This concept is supported by the following evidence: (1) overexpression of ephrinB1or Dsh2 or PKCδ in the D1.1.1 blastomere does not rescue Sprouty2 MO-induced inhibition of retinal population (Fig. 2a,b and Supplementary Fig. 5a). Similarly, neither Sprouty2 nor DN-Raf overexpression in the D1.1.1 blastomere restores retinal population in the absence of ephrinB1(Supplementary Fig. 6b); (2) overexpression of PKCδ in the D1.1.1 blastomere did not affect the level of MAPK phosphorylation (Supplementary Fig. 5b), suggesting that the ephrinB1/Dsh2-mediated PCP pathway does not function upstream of the MAPK pathway; (3) overexpression of ephrinB1 in the ventral V1.1.1 blastomere, whose progeny contributes little (< 1%) to retina, can drive the movement of ventral epidermal progenitors into eye field. However, neither Sprouty2 nor DN-Raf overexpression promotes the movement of V1.1.1 progeny into the retinal field (Supplementary Fig. 6a); (4) It has been shown that blocking of ephrinB1/Dsh2-mediated PCP signaling restricts D1.1.1 clones from dispersing outside the midline during gastrulation^9^. In contrast, loss of Sprouty2 does not affect the movement of D1.1.1 clones until the late neurula stage, but not earlier stages (Fig. 4a and Supplementary Fig. 1), which is consistent with the Sprouty2 expression pattern (Fig. 1a). Thus, Sprouty2-mediated Ras/Raf/MAPK signaling functions independent of the ephrinB1/Dsh2-mediated PCP pathway, although both contribute to retina formation.

The Ras/Raf/MAPK signaling pathway has been shown to function as a potential inducer of the Epithelial–Mesenchymal Transition (EMT)^31–33^. EMT is an important process during embryonic development and tumor progression, in which epithelial cells acquire mesenchymal, fibroblast-like properties and show reduced cell adhesion and increased motility^34–37^. It has been reported that activated MAPK/ERK promotes invasion and metastasis of different cancer cells by triggering EMT^38,39^. The eye field that consists of retina progenitors is derived from neuroepithelium, whose movement is regulated by the PCP signaling pathway^9,40–42^. It is important that the retina progenitors maintain a low level of MAPK activity to inhibit EMT which could induce loss of cell junctions, polarity, and promote retina progenitors to escape the control of the PCP signal. In this study, we show that low level of MAPK activity is normally required for retinal progenitors positioning within the optic vesicle at the late neurula stage, and enhanced MAPK signaling prevents these progenitors from populating the retina.

In summary, our findings demonstrate that Sprouty2 regulates positioning of retinal progenitors through suppressing of Ras/Raf/MAPK pathway.

## Methods

### Embryo, morpholino and microinjection

Xenopus embryos were obtained by standard methods^43^. Capped mRNAs for microinjection were synthesized with the SP6 mMessage mMachine Kit (Invitrogen). Embryos were injected at 32 cell stage into D1.1.1 blastomere with the following mRNAs: *GFP* (120 pg); *sprouty2 *(Accession number: NM_001088769) (200 pg); *sprouty2*^*YF*^ (200 pg); *sprouty2 MOR* (200 pg); *ephrinB1 *(Accession number: *NM_001087479*) *MOR* (200 pg); *XDsh2 *(*Accession number:* NM_001090627) *MOR* (250 pg); *DN-Ras *(*Accession number:* NM_001130443) (200 pg); *DN-Raf* (Accession number: NM_001088006) (200 pg); *ca-FgfR2 *(*Accession number:* NM_001090663) (50 pg); *ca-Raf* (40 pg). The morpholinos were obtained from Gene Tools with the following sequences: Sprouty2 MO: 5′-TACTCTGAAGAGTTTTCACACCAGT-3′, ephrinB1 MO: 5′-GGAGCCCTTCCATCCGCACAGGTGG-3′, XDsh2 MO: 5′-TCACTTTAGTCTCCGCCATTCTGCG-3′, PKCδ MO: 5′-AGGATATGCGTAGGAAGGAGACATG-3′, control MO: 5′-CTAAACTTGTGGTTCTGGCGGATA-3′.

### Whole-mount in situ hybridization

Embryos were injected with *GFP* RNA and various RNA or MOs and GFP expression was used to distinguish the injected side of stage 18 embryos, and then processed for whole-mount in situ hybridization by using standard methods with probes for rx1and pax6^9^. For RNA in situ probe against Xsprouty2 was generated by linearizing with Bam HI and transcribing with T7.

### Histology and immunohistochemistry

For immunofluorescence assay on sections, the embryos were collected at stage 33 and fixed in 4% paraformaldehyde in PBS overnight at 4 °C. embryos were then embedded in 4% low melting agarose gel and were sectioned with a thickness of 50–60 μm with the vibratome (LEICA VT 1200S). The primary antibodies used were: Chicken anti-GFP (Abcam), rabbit anti-Cleaved Caspase-3 (Cell Signaling Technology), rabbit anti-phospho-histone 3 (Millipore). The secondary antibodies used were Alexa Fluor-488 or Alexa Fluor-594 conjugated Goat anti-rabbit IgG or anti-chicken IgG (Invitrogen). The samples were washed, mounted and imaged using an Zeiss LSM710 laser scanning confocal microscope.

### Immunoprecipitation and Western blot analysis

The process of Immunoprecipitation was previously described^44^. Briefly, *Xenopus* embryo lysates were prepared with ice-cold TNSG buffer (20 mM Tris–HCl pH 7.5, 137 mM NaCl and 1% NP-40). IPs were performed for 4 h with 25 embryo equivalent extracts using monoclonal Anti-V5-agarose (Sigma-Aldrich). Western blot analysis was performed using anti-Flag–horseradish peroxidase (HRP)-conjugated (1:5,000, Sigma-Aldrich), anti-HA–HRP-conjugated (1:5,000, Sigma-Aldrich) anti-V5–HRP-conjugated (1:5,000, Sigma-Aldrich), anti-Phospho-p44/42 MAPK (Erk1/2) (Cell Signaling Technology), and anti-p44/42 MAPK (Erk1/2) (Cell Signaling Technology) antibodies.

### U0126 treatment

Embryos were injected with control MO or sprouty2 MO into the D1.1.1 blastomere at 32 cell stage. U0126, a MEK1/2 inhibitor were applied to the embryo culture medium from stage 12 to stage 25. Embryos were then fixed and prepared for section immunostaining at stage 33.

### Statistics

The one-way ANOVA with multiple comparisons test or t test was performed using Prism8.

### Ethics statement

Animal care and use for this study were performed in accordance with the recommendations of AAALAC for the care and use of laboratory animals in an AAALAC approved facility. Experimental procedures were specifically approved by the animal care & use committee of the of the National Cancer Institute-Frederick ASP #18-433 in compliance with AAALAC guidelines. J.S., J.Y., M.L., Y.-S.H., I.O.D. all possess the authorization for vertebrates’ experimental use.

## Supplementary information

Supplementary Figures.
