# Supplementary material for: Sprouty2 regulates positioning of retinal progenitors through suppressing the Ras/Raf/MAPK pathway
Source: Sci Rep. 2020 Aug 13;10:13752. doi: 10.1038/s41598-020-70670-2 (PMC7426826; doi:10.1038/s41598-020-70670-2)
Supplement: Supplementary file 1 — Supplementary Figures. [file 41598_2020_70670_MOESM1_ESM.pdf]

Sprouty2 regulates positioning of retinal progenitors through suppressing the Ras/Raf/MAPK pathway

Jian Sun[1], Jaeho Yoon[1], Moonsup Lee[1], Yoo-Seok Hwang[1] and Ira O. Daar[1*]

[1]Cancer & Developmental Biology Laboratory, National Cancer Institute, Frederick, Maryland 21702, USA

[*]Correspondence should be addressed to I.O.D. (e-mail: daari@mail.nih.gov)

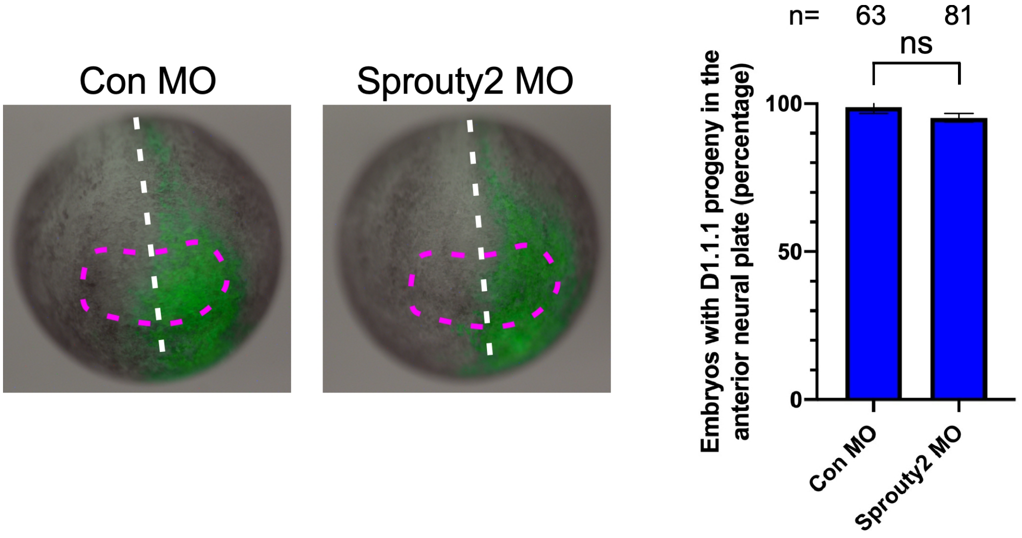

Supplementary Figure 1. Sprouty2 knockdown does not affect retinal progenitors movement into eye field at early neurula stage.

D1.1.1 blastomere was injected with GFP RNA plus control MO or Sprouty2 MO as indicated. Embryos were harvested at stage 15. Both control embryo and Sprouty2 morphant showed normal dispersion of D1.1.1 clones in the eye field that was outlined with purple dotted line. The white dotted line indicates the embryo midline. Histograms represent the percentage of embryos with D1.1.1 progeny (GFP positive signals) within the eye field from three biological repeats. Quantification with unpaired t test, $P > 0.05$, Error bars indicate ± SD. ns: no statistical differences between groups.

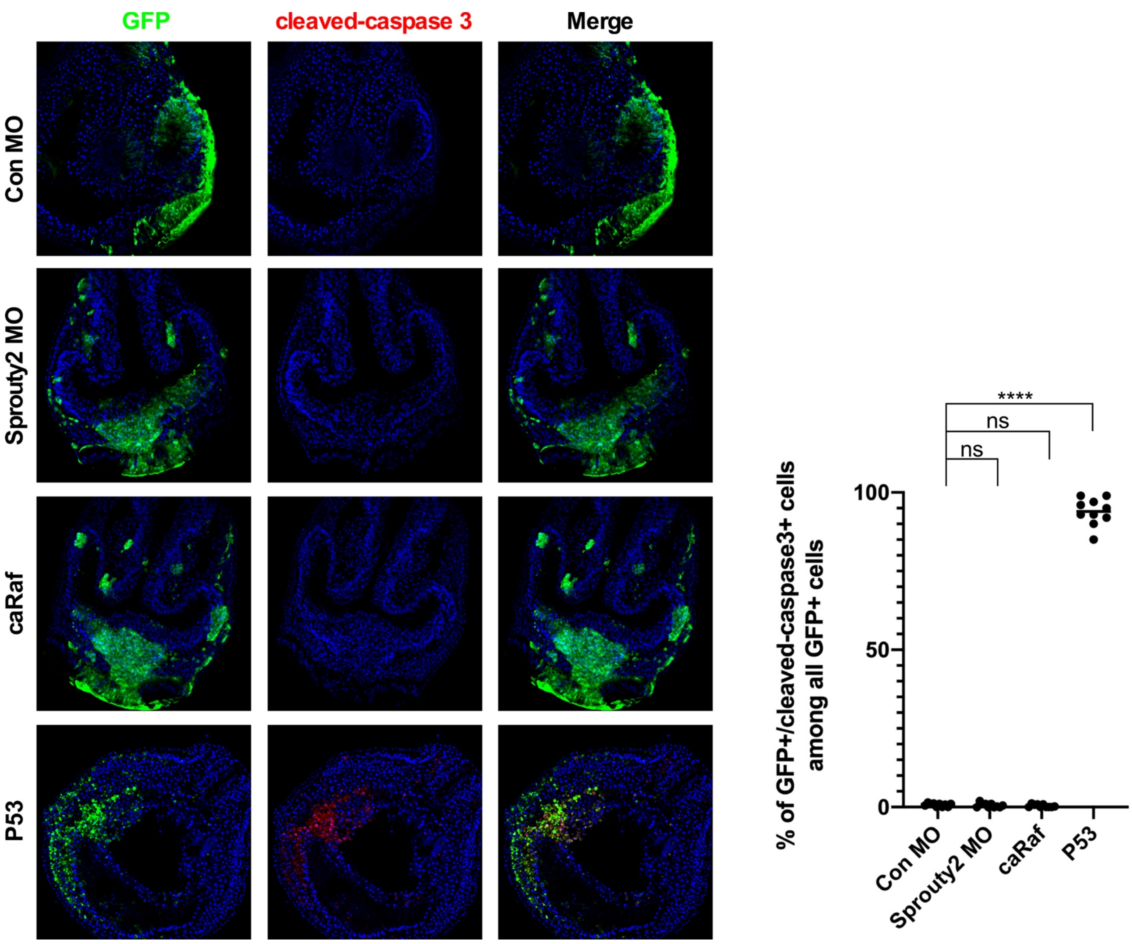

Supplementary Figure 2. Knockdown of Sprouty2 does not induce apoptosis in D1.1.1 progeny at late neurula stage.

Indicated morpholinos or RNAs were injected with GFP RNA into D1.1.1 blastomere at 32 cell stage. Embryos were sectioned and immunostained with cleaved-caspase 3 antibody at stage 19. Scatterplots represent cleaved-caspase 3+ cells expressing GFP on total GFP+ cells from three biological experiments. Quantification with one-way ANOVA (Dunnett's multiple comparison), $P < 0.0001$. Error bars indicate ± SD. ****$P < 0.0001$, ns: no statistical differences between groups.

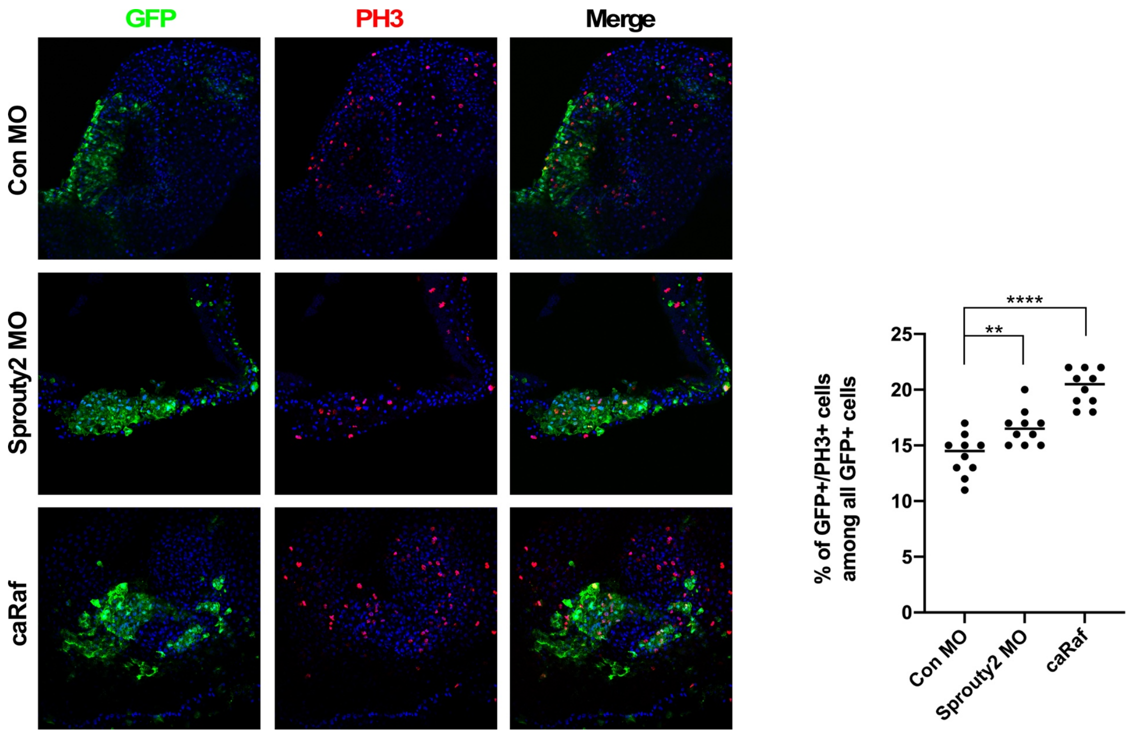

Supplementary Figure 3. Knockdown of Sprouty2 slightly increases proliferation in D1.1.1 progeny at late neurula stage.

Indicated morpholinos or RNAs were injected with GFP RNA into D1.1.1 blastomere at 32 cell stage. Embryos were sectioned and immunostained with phospho-Histone 3 (PH3) antibody at stage 19. Scatterplots represent PH3+ cells expressing GFP on total GFP+ cells from three biological experiments. Quantification with one-way ANOVA (Dunnett's multiple comparison), $P < 0.0001$. Error bars indicate ± SD. $^{**}P < 0.01$; $^{****}P < 0.0001$; ns: no statistical differences between groups.

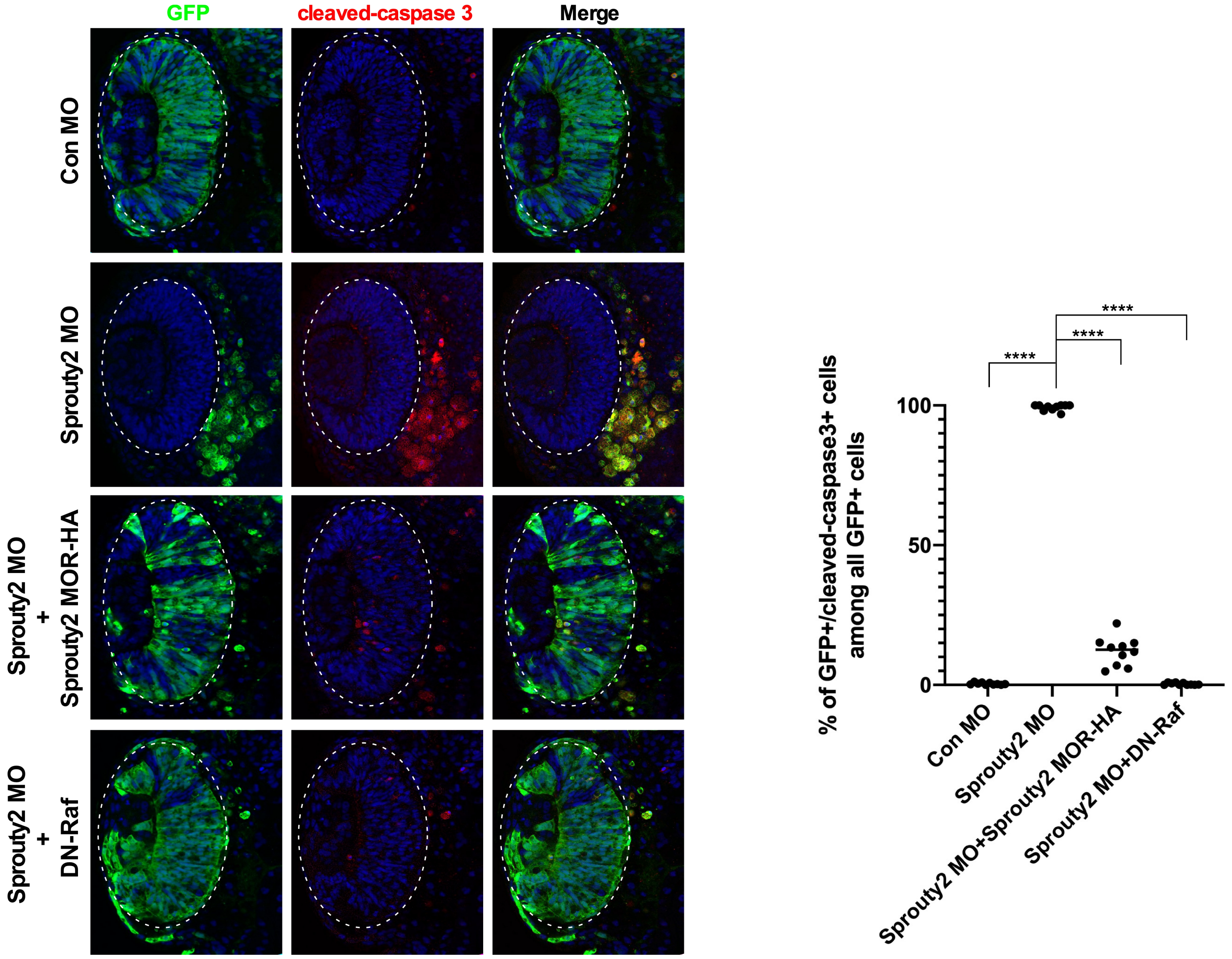

Supplementary Figure 4. Depletion of Sprouty2 prevents D1.1.1 progeny from populating retina and induces cell apoptosis at tadpole stage.

Indicated morpholinos or RNAs were injected with GFP RNA into D1.1.1 blastomere at 32 cell stage. Embryos were sectioned and immunostained with cleaved-caspase 3 antibody at stage 33. The retina was outline with oval dotted line. Scatterplots represent cleaved-caspase 3+ cells expressing GFP on total GFP+ cells from three biological experiments. Quantification with one-way ANOVA (Dunnett's multiple comparison), $P < 0.0001$. Error bars indicate ± SD. ****$P < 0.0001$, ns: no statistical differences between groups.

a

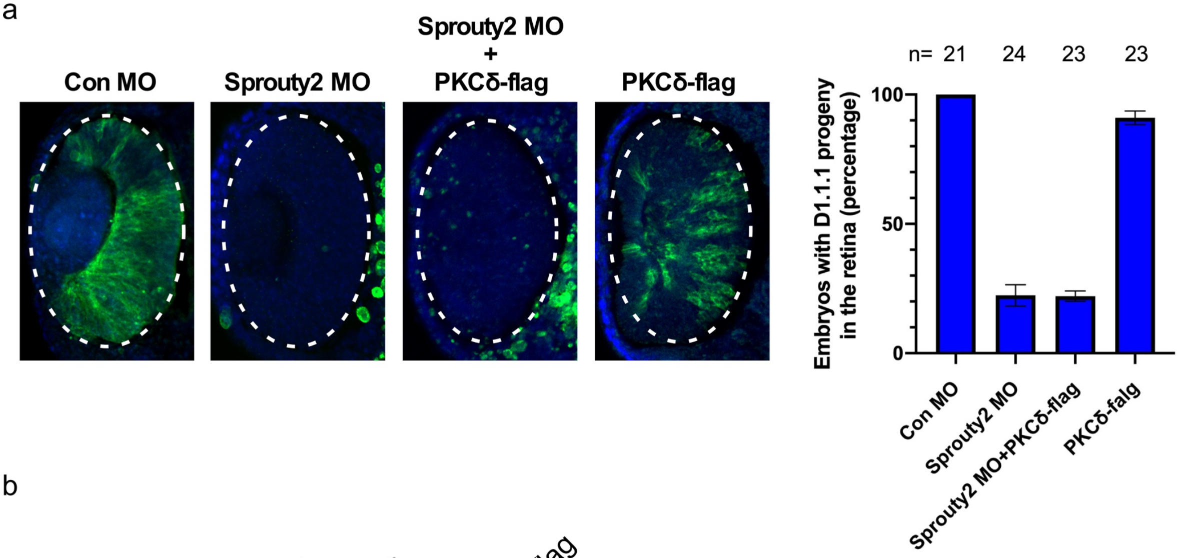

b

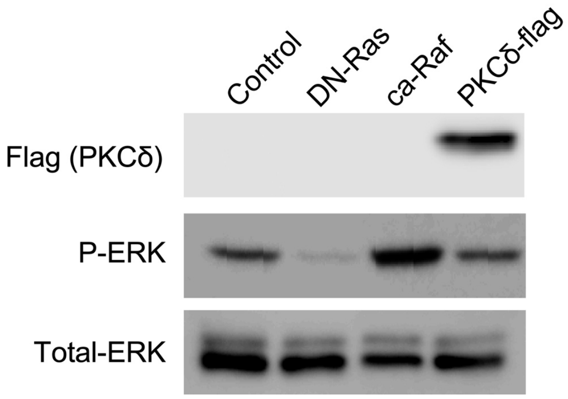

Supplementary Figure 5. PKCδ is not involved in the regulation of Sprouty2-mediated retina population.

(**a**) Indicated morpholinos or RNAs were injected with GFP mRNA into D1.1.1 blastomere at 32 cell stage. Embryos then were sectioned and immunostained with GFP antibody (Green) at stage 33. Images were taken on the eye region. The retina was outline with oval dotted line. Histograms represent the percentage of embryos with D1.1.1 progeny (GFP positive signals) within the retina from three biological repeats. Quantification with one-way ANOVA (Sidak's multiple comparison), $P < 0.0001$. (**b**) Embryos were injected with RNAs or Morpholinos as indicated at one cell stage. Injected embryos were lysed at gastrula stage and then immunoblotted with phospho-ERK antibody and total ERK antibody. Three independent repeats were performed.

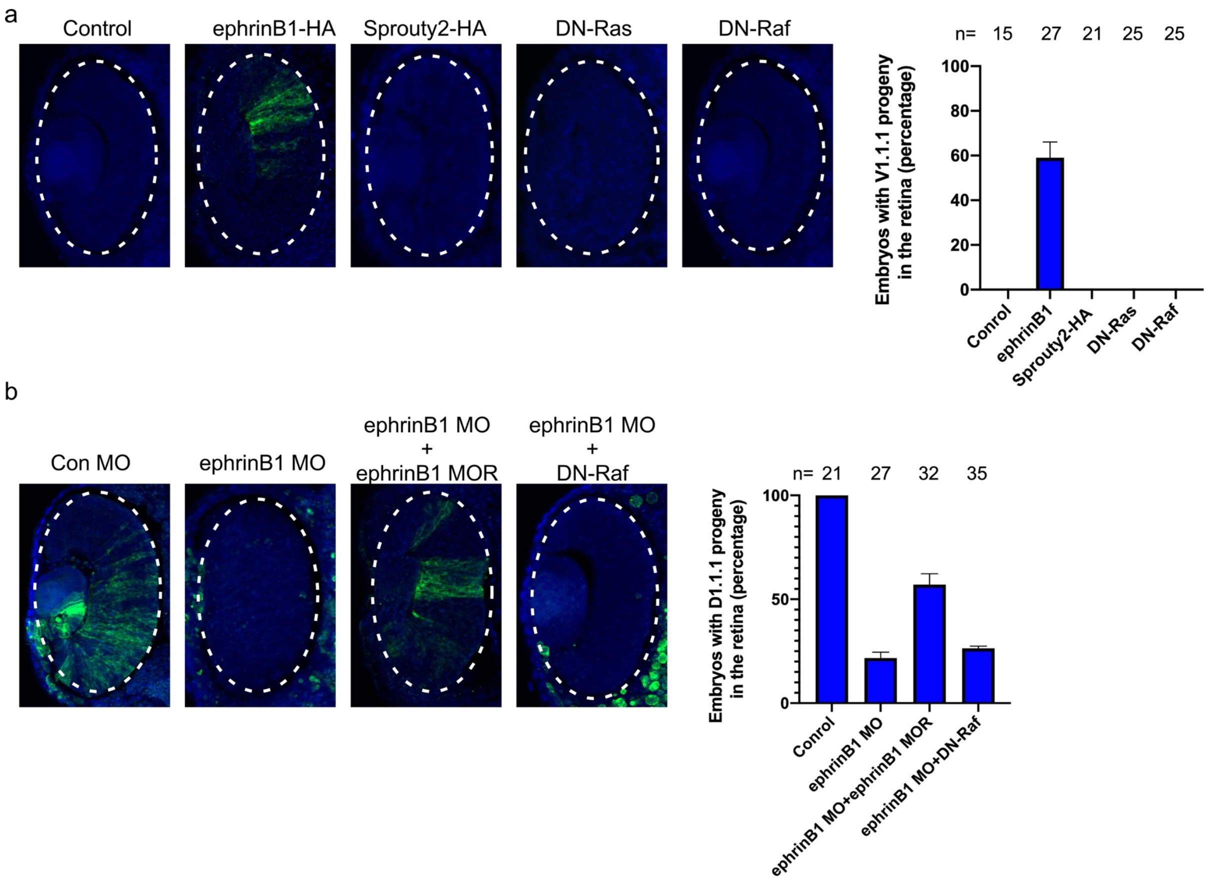

Supplementary Figure 6. Sprouty2 is not involved in the regulation of ephrinB1-mediated retina population.

(**a**) Indicated morpholinos or RNAs were injected with GFP mRNA into V1.1.1 blastomere at 32 cell stage. Embryos then were sectioned and immunostained with GFP antibody (Green) at stage 33. Images were taken on the eye region. The retina was outline with oval dotted line. Histograms represent the percentage of embryos with V1.1.1 progeny (GFP positive signals) within the retina from three biological repeats. Quantification with one-way ANOVA (Sidak's multiple comparison), $P < 0.0001$. (**b**) Indicated morpholinos or RNAs were injected with GFP mRNA into D1.1.1 blastomere at 32 cell stage. Histograms represent the percentage of embryos with D1.1.1 progeny (GFP positive signals) within the retina from three biological repeats. Quantification with one-way ANOVA (Sidak's multiple comparison), $P < 0.0001$.

a

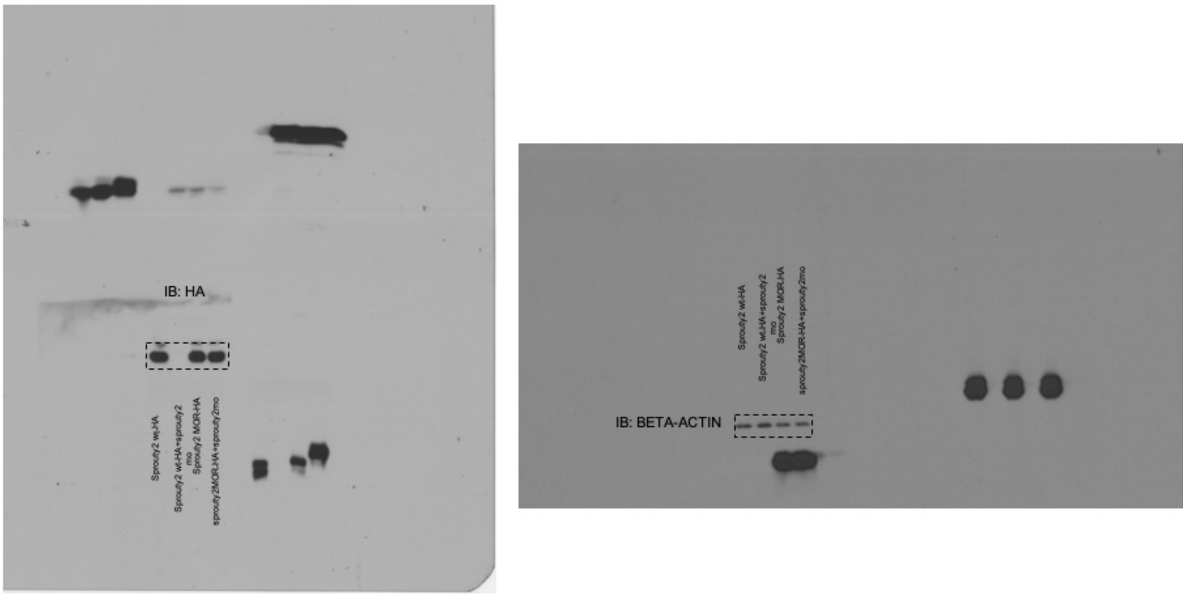
IB: HA
Sprouty2 wt-HA
Sprouty2 wt-HA+sprouty2 mo
Sprouty2 MOR-HA
sprouty2MOR-HA+sprouty2mo
Sprouty2 wt-HA
Sprouty2 wt-HA+sprouty2 mo
Sprouty2 MOR-HA
sprouty2MOR-HA+sprouty2mo
IB: BETA-ACTIN

b

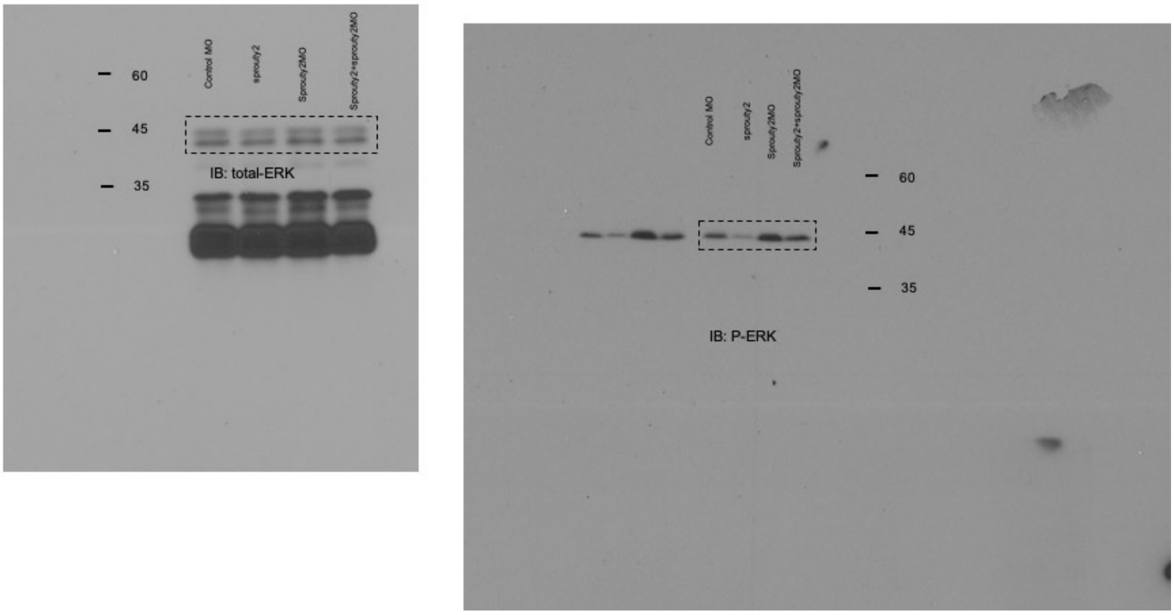
60
45
35
Control MO
sprouty2
Sprouty2MO
Sprouty2+sprouty2MO
IB: total-ERK
Control MO
sprouty2
Sprouty2MO
Sprouty2+sprouty2MO
60
45
35
IB: P-ERK

c

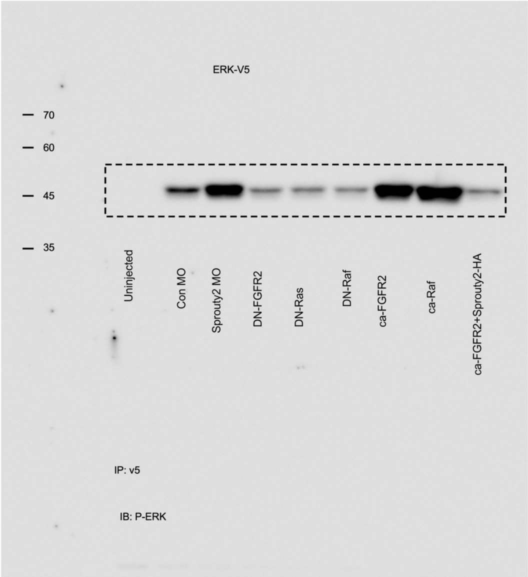
ERK-V5
70
60
45
35
Uninjected
Con MO
Sprouty2 MO
DN-FGFR2
DN-Ras
DN-Raf
ca-FGFR2
ca-Raf
ca-FGFR2+Sprouty2-HA
IP: v5
IB: P-ERK

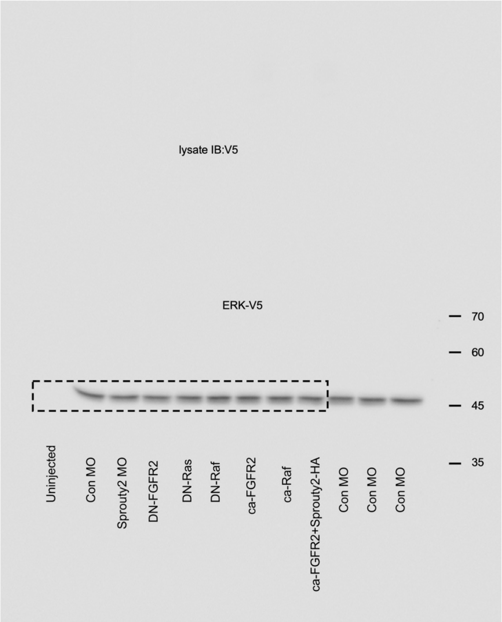
lysate IB:V5
ERK-V5
70
60
45
35
Uninjected
Con MO
Sprouty2 MO
DN-FGFR2
DN-Ras
DN-Raf
ca-FGFR2
ca-Raf
ca-FGFR2+Sprouty2-HA
Con MO
Con MO
Con MO

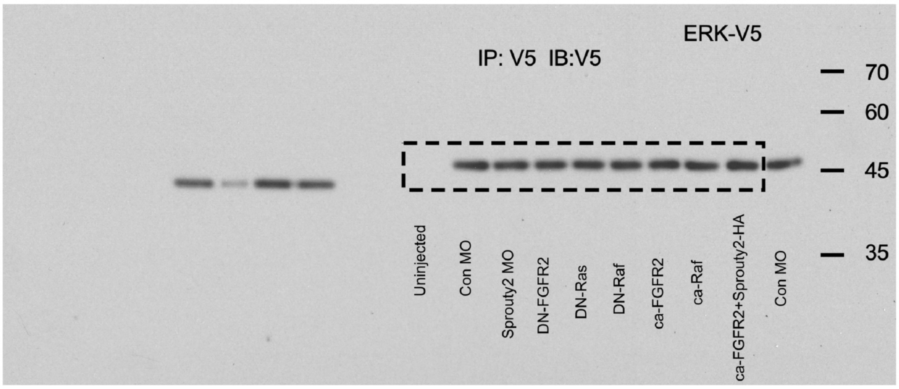
ERK-V5
IP: V5 IB:V5
70
60
45
35
Uninjected
Con MO
Sprouty2 MO
DN-FGFR2
DN-Ras
DN-Raf
ca-FGFR2
ca-Raf
ca-FGFR2+Sprouty2-HA
Con MO

d

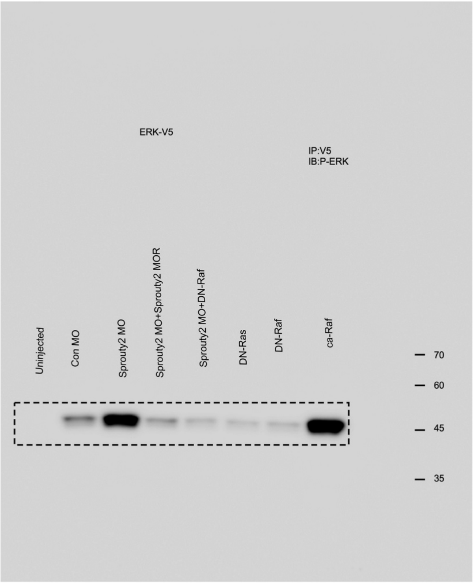
ERK-V5
IP:V5
IB:P-ERK
Uninjected
Con MO
Sprouty2 MO
Sprouty2 MO+Sprouty2 MOR
Sprouty2 MO+DN-Raf
DN-Ras
DN-Raf
ca-Raf
70
60
45
35

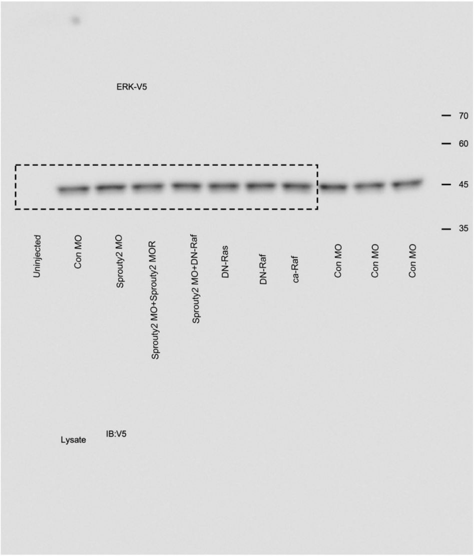
ERK-V5
70
60
45
35
Uninjected
Con MO
Sprouty2 MO
Sprouty2 MO+Sprouty2 MOR
Sprouty2 MO+DN-Raf
DN-Ras
DN-Raf
ca-Raf
Con MO
Con MO
Con MO
Lysate
IB:V5

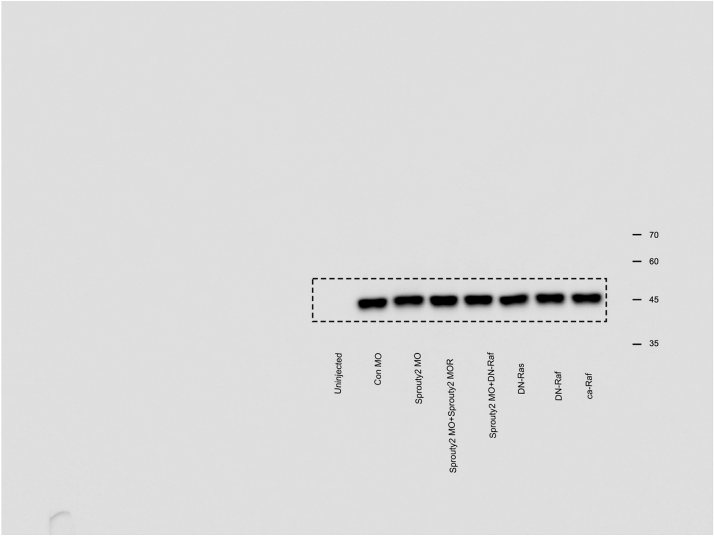
70
60
45
35
Uninjected
Con MO
Sprouty2 MO
Sprouty2 MO+Sprouty2 MOR
Sprouty2 MO+DN-Raf
DN-Ras
DN-Raf
ca-Raf

e

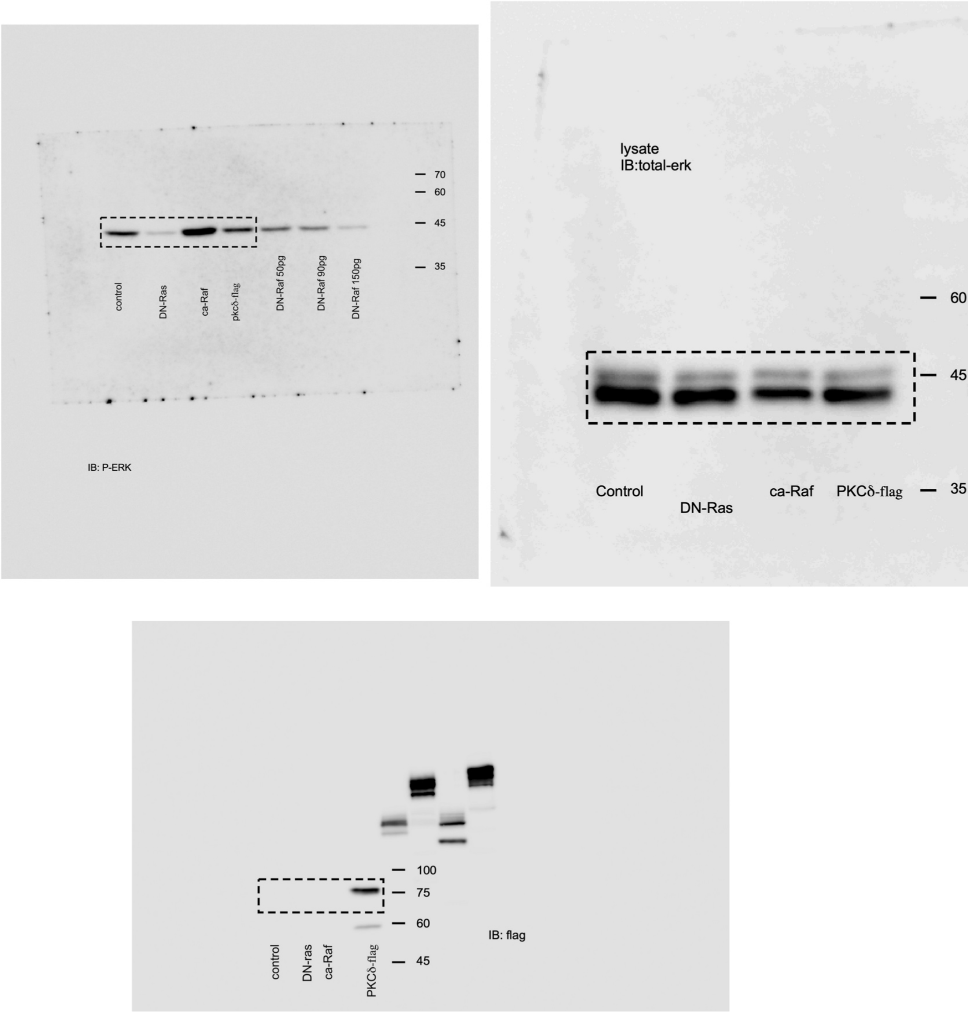

Supplementary Figure 7. Uncropped images.

(**a**) Figure 1b. (**b**) Figure 1c. (**c**) Figure 3b. (**d**) Figure 4b. (**e**) Supplementary Figure 5b. Dashed boxes indicate the portion of gels included in the figures.

.
